# Comparative Analysis of High-Frequency and Low-Frequency Transcutaneous Electrical Stimulation of the Right Median Nerve in the Regression of Clinical and Neurophysiological Manifestations of Generalized Anxiety Disorder

**DOI:** 10.3390/jcm13113026

**Published:** 2024-05-21

**Authors:** Mustafa Al-Zamil, Natalia G. Kulikova, Inessa A. Minenko, Irina P. Shurygina, Marina M. Petrova, Numman Mansur, Rufat R. Kuliev, Vasilissa V. Blinova, Olga V. Khripunova, Natalia A. Shnayder

**Affiliations:** 1 Department of Physiotherapy, Faculty of Continuing Medical Education, Peoples’ Friendship University of Russia, 117198 Moscow, Russia; kulikovang777@mail.ru (N.G.K.); d-64-158@mail.ru (N.M.); vasilissablinova@yandex.ru (V.V.B.); 2 Department of Sports Medicine and Medical Rehabilitation, I.M. Sechenov First Moscow State Medical University, 119991 Moscow, Russia; kuz-inna@mail.ru (I.A.M.); olaw@bk.ru (O.V.K.); 3 Department of Restorative Medicine and Neurorehabilitation, Medical Dental Institute, 127253 Moscow, Russia; roofik-92@mail.ru; 4 Department of Ophthalmology, Rostov State Medical University, 344022 Rostov, Russia; ir.shur@yandex.ru; 5 Shared Core Facilities “Molecular and Cell Technologies”, Professor V. F. Voino-Yasenetsky Krasnoyarsk State Medical University, 660022 Krasnoyarsk, Russia; stk99@yandex.ru; 6 City Clinical Hospital Named after V. V. Vinogradov, 117292 Moscow, Russia; 7 Institute of Personalized Psychiatry and Neurology, V.M. Bekhterev National Medical Research Centre for Psychiatry and Neurology, 192019 Saint Petersburg, Russia

**Keywords:** transcutaneous electrical nerve stimulation, generalized anxiety disorder, HF-TENS, LF-TENS, GAD-7, HAM-A, coefficient coherence, power spectral density

## Abstract

**Background/Objectives**: The anxiolytic effect of transcutaneous electrical nerve stimulation (TENS) is associated with the activation of endogenous inhibitory mechanisms in the central nervous system. Both low-frequency, high-amplitude TENS (LF-TENS) and high-frequency, low-amplitude TENS (HF-TENS) are capable of activating opioid, GABA, serotonin, muscarinic, and cannabinoid receptors. However, there has been no comparative analysis of the effectiveness of HF-TENS and LF-TENS in the treatment of GAD. The purpose of our research was to study the effectiveness of direct HF-TENS and LF-TENS of the right median nerve in the treatment of patients with GAD compared with sham TENS. **Methods:** The effectiveness of direct HF-TENS and LF-TENS of the right median nerve in the treatment of GAD was studied using Generalized Anxiety Disorder 7-item scale (GAD-7) and the Hamilton Anxiety Rating Scale (HAM-A). 40 patients underwent sham TENS, 40 patients passed HF-TENS (50 Hz—50 μs—sensory response) and 41 patients completed LF –TENS (1 Hz—200 μs—motor response) for 30 days daily. After completion of treatment, half of the patients received weekly maintenance therapy for 6 months. Electroencephalography was performed before and after treatment. **Results:** Our study showed that a significant reduction in the clinical symptoms of GAD as assessed by GAD-7 and HAM-A was observed after HF-TENS and LF-TENS by an average of 42.4%, and after sham stimulation only by 13.5% for at least 2 months after the end of treatment. However, LF-TENS turned out to be superior in effectiveness to HF-TENS by 51% and only on electroencephalography leads to an increase in PSD for the alpha rhythm in the occipital regions by 24% and a decrease in PSD for the beta I rhythm in the temporal and frontal regions by 28%. The prolonged effect of HF-TENS and LF-TENS was maintained without negative dynamics when TENS treatment was continued weekly throughout the entire six-month observation period. **Conclusions:** A prolonged anxiolytic effect of direct TENS of the right median nerve has been proven with greater regression of clinical and neurophysiological manifestations of GAD after LF-TENS compared to HF-TENS. Minimal side effects, low cost, safety, and simplicity of TENS procedures are appropriate as a home treatment modality.

## 1. Introduction

Generalized Anxiety Disorder (GAD) (ICD-10: F41.1) is a mental health disorder that is characterized by excessive, uncontrollable, and often irrational worry, that is, apprehensive expectations about events or activities [1]. This excessive worry often interferes with daily functioning [2]. The psychological portrait is characterized by pessimism, panophobia, and inability to relax [3]. The relevance of this disease lies in the development of secondary somatic symptomatology such as fibromyalgia, irritable bowel syndrome, and many other diseases, which may be at the forefront while GAD remains undiagnosed for a long time [4]. Currently, the relevance of this disease has increased; since during COVID-19, the incidence of GAD has become 6 times more common (30.5%) [5] compared to the pre-COVID-19 pandemic (5%) [6,7].

According to the *Diagnostic and Statistical Manual of Mental Disorders, Fifth Edition* (DSM-5) published by the American Psychiatric Association in 2013 in GAD, anxiety and worry must be associated with three (or more) of the following six symptoms: restlessness or feeling keyed up or on edge, being easily fatigued, difficulty concentrating or mind going blank, irritability, muscle tension, and sleep disturbance for at least 6 months [8].

Meta-analytical integrations of family studies revealed a genetic heritability of 31.6–67% [9,10]. Other genetic studies found that lifetime diagnosis of GAD is associated with intronic rs78602344 polymorphism of the throm-bospondin-2 gene (THBS2) in chromosome 6 [11], intronic rs1709393 minor C allele polymorphism on chromosomal band 3q12.3 [12], and intronic rs1067327 polymorphism on chromosome 2p21 [12].

Genetic polymorphisms are associated with cerebral activity in the amygdala and hippocampus or connectivity between the right amygdala and the fusiform gyrus, as shown in a series of functional magnetic resonance imaging (fMRI) studies. In addition, an increase in cortical activity and a decrease in basal ganglia activity were found, which reversed with treatment [13]. Furthermore, brain signal variability has been shown to be reduced in patients with GAD in widespread regions, including the visual network, sensorimotor network, frontoparietal network, limbic system, and thalamus, suggesting an inflexible brain state transfer pattern in these systems [14].

Clinically, electroencephalography (EEG) has been used to diagnose GAD for more than 20 years [15]. By analyzing multivariate EEG features such as univariate power spectral density (PSD), fuzzy entropy (FE), and multivariate functional connectivity (FC), patterns of this disease were discovered [16]. According to many researchers, the EEG pattern in GAD represents a significant increase in the general beta rhythm >17% and high beta waves in the temporal regions (T3 and T4) >10%, simultaneously with a reliable decrease in the alpha rhythm PSD. As a result, a noticeable decrease in long-range FC develops between the frontal and other regions of the brain in all frequency ranges [16,17,18,19,20].

Optimal treatment of GAD must include both pharmacologic and behavioral interventions [21]. Although a range of pharmacological agents are available to treat GAD, it is estimated that up to half of patients receiving treatment did not respond adequately to the chosen therapy [22]. Moreover, a prolonged course of the disease can cause addiction to pharmacotherapy, drug resistance, or the development of side effects [23]. In this regard, there is a need to use additional non-drug therapy to enhance the therapeutic effect in the treatment of patients with GAD [24,25].

Transcutaneous electrical nerve stimulation (TENS) has analgesic, restorative, and regenerative effects [26,27,28]. However, the anxiolytic effect of TENS has not been well studied [29]. Nonetheless, several studies have been published demonstrating the effectiveness of TENS in the treatment of anxiety disorders during and after the procedure [26,28,30,31].

The anxiolytic effect of TENS is associated with the activation of endogenous inhibitory mechanisms in the central nervous system.

Both LF-TENS and HF-TENS are capable of activating opioid, GABA, serotonin, muscarinic, and cannabinoid receptors [32]. Specifically, LF-TENS activates mu-opioid receptors, and HF-TENS activates δ-opioid receptors [33]. In addition, HF-TENS and LF-TENS increase the level of B-endorphin in the blood plasma [34], cerebrospinal fluid [35], and the bloodstream [32] and methionine-enkephalin in the cerebrospinal fluid [36].

TENS along with transcranial magnetic stimulation (TMS) and transcranial direct current stimulation (tDCS) have proven to be promising therapeutic modalities for the treatment of anxiety and depression by modulating neuroplasticity by inducing changes in cortical excitability and connectivity [37,38]. Using fMRI, HF-TENS was found to decrease pontine activity and connectivity between the pons and somatosensory cortex, whereas LF-TENS increased functional connectivity in the medulla and decreased functional connectivity between the frontal cortex and medulla [38]. Recently, many authors have suggested that the amygdala is a key emotion-processing region. Functional connectivity deficits in the amygdala and frontal region can be modulated using TENS. Resting-state fMRI scanning was used before and after TENS treatment. After a month of TENS use, fMRI revealed increased resting-state functional connectivity of the amygdala-lateral prefrontal network, which correlated with decreased scores of anxiety and depression [39]. Other studies have found that TENS, in addition to the amygdala, can modulate other cortical and subcortical structures such as the locus coeruleus, anterior cingulate cortex, and medial prefrontal cortex, which play a crucial role in the emotional regulation network [40]. However, decreased cortical activity in the somatosensory and motor cortices has been documented by EEG during both LF-TENS and HF-TENS [41,42].

Additionally, TENS has a beneficial effect on the autonomic nervous system. This effect was observed when sympathetic activity was suppressed in patients with arterial hypertension after direct stimulation of the median nerve in different frequency ranges [43]. Immunohistochemical studies have shown that TENS increases the expression of oxytocin and decreases the expression of corticotropin-releasing factor in the paraventricular nucleus of the hypothalamus [44,45]. Many studies have shown that hypothalamic oxytocin has anti-stress effects by inhibiting the expression of corticotropin-releasing factor and the activity of the hypothalamic–pituitary–adrenal axis [46,47].

One of the most relevant anxiolytic mechanisms of TENS is the reduction in inflammatory cytokines, which are classically associated with increased oxidative stress and sympathetic activity in patients with anxiety disorders [48].

Prior to this work, no studies had examined the effectiveness of direct TENS in the treatment of GAD. No comparison has been made between the effectiveness of HF-TENS and LF-TENS. In this regard, the aim of our research was to study the effectiveness of direct HF-TENS and LF-TENS of the right median nerve in the treatment of patients with GAD compared to sham TENS.

## 2. Materials and Methods

### 2.1. Study Design and Population

In this single-center randomized sham-controlled trial, we observed 428 patients with GAD.

Inclusion criteria in the study:

1. European;
2. adult men and women from 25 to 60 years old;
3. history of at least 3 symptoms of GAD: apprehension, motor tension, and autonomic overactivity;
4. GAD older than 6 months but less than 3 years;
5. the severity of GAD by GAD-7 is mild and moderate (5–14 points);
6. non-smokers;
7. passed ineffective pharmacotherapy or treatment was canceled due to side effects of the drugs at least 3 months before the start of the study;
8. signed voluntary informed consent to participate in this study.

Exclusion criteria in the study:

1. epilepsy and uncontrolled seizure disorder;
2. severe cognitive disorders;
3. damage to the median nerve anywhere along its path from the brachial plexus to the carpal canal;
4. pregnancy;
5. history of stroke, spinal cord injury, traumatic brain injury, multiple sclerosis, edema of upper extremity;
6. history of cardiac arrhythmias or hemodynamic instability;
7. cardiac pacemaker or other implanted electronic system;
8. history of obsessive-compulsive disorder;
9. patients without stable housing (locator person);
10. out of work and experiencing significant financial difficulties;
11. shift and night shift jobs;
12. alcohol and drug use disorder;
13. late-night gaming;
14. frequent consumption of coffee and caffeinated drinks;
15. taking tranquilizers and sedative and psychotropic drugs during treatment;
16. undergoing physiotherapy or acupuncture treatment.

Voluntary Informed Consent in Research and Clinical Care was signed by all patients, after an explanation of the medical condition, the purpose and benefits of the test, procedure, or treatment and a description of the proposed test, procedure, or treatment, including possible complications or adverse events. The study protocol was approved by the local Ethics Medical Committee of Peoples’ Friendship University of Russia, 117198 Moscow, Russia (protocol No. 128, 2 December 2022). All procedures adhered to the 1984 Declaration of Helsinki and its later amendments. All patients have read the above-named article in full (including text and figures, and agree to its publication).

Participation is not rewarded in this study. The researchers were not rewarded for their work. The study was carried out as part of a scientific research program of the Department of Physiotherapy of Peoples’ Friendship University of Russia.

**Figure 1:**
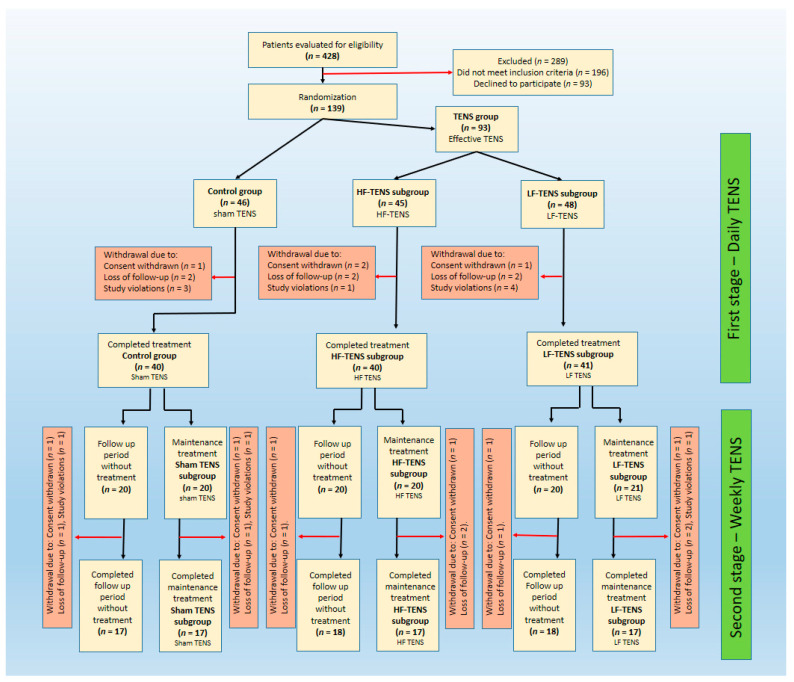
Flow chart of study population selection. Note: Sham TENS: sham transcutaneous electrical nerve stimulation; HF-TENS: high-frequency, low-amplitude transcutaneous electrical nerve stimulation; LF-TENS: low-frequency, high-amplitude transcutaneous electrical nerve stimulation.

Four hundred and twenty-eight patients (female—225, male—203) with GAD were eligible to participate in this research. Two hundred and eighty-nine of them did not meet the inclusion criteria or refused further participation. One hundred and thirty-nine patients (female—67, male—72) meeting all inclusion criteria were randomized in a 1:1:1 ratio. Eighteen patients discontinued participation due to withdrawal of consent, loss of follow-up, and study violations, reducing the total number of patients to 121 (female—58, male—63). The control group included 40 patients (female—19, male—21) who received only sham TENS. Effective TENS was carried out in the treatment group, which was divided into 2 subgroups in accordance with the characteristics of the performed TENS. First subgroup which consisted of 40 patients (female—19, male—21) underwent HF-TENS (HF-TENS subgroup). In the second subgroup, consisting of 41 patients (female—20, male—21), LF-TENS was used (LF-TENS subgroup). The female/male ratios were comparable in the control group, in the treatment group, and in all TENS subgroups (Figure 1).

Our study consisted of two stages. In the first stage, we studied the effectiveness of TENS when used every day for 30 days (daily TENS). In the second stage, approximately half of the patients received maintenance TENS once a week for 6 months after completion of the first stage (weekly TENS).

Demographic data and clinical characteristics of the participants are summarized in Table 1. The age of the participants ranged from 28 to 58 years and averaged 46.2 ± 0.62 years. The male-to-female ratio of the study participants, control group, and all TENS subgroups was 1:1. In the total sample, patients suffered from GAD symptoms for 7 to 34 months (mean duration—18.2 ± 0.74 months). On the GAD-7 scale, the severity of anxiety disorders was assessed from 6 to 14 points, with an average score of 10.62 ± 0.18 points. The results of the HAM-A anxiety assessment for all participants ranged from 8 to 23 points and averaged 15.14 ± 0.31 points. Between-group differences in patient number, age, gender ratio, GAD duration, and severity of GAD symptoms assessed by GAD-7 and HAM-A were not statistically significant (*p*-value > 0.05).

### 2.2. Sample Size Calculation

To determine the minimum number of subjects in each group, we used the sample size calculator at https://clincalc.com/stats/samplesize.aspxsite (accessed on 5 March 2022). A literature search revealed that in a study of the anxiolytic effects of transcranial direct current stimulation in the treatment of patients with GAD, HAM-A scores (M ± SD) decreased in the treatment group from 44.3 ± 5.5 to 35.3 ± 3.57 by 20.3% and did not change in the control group after sham stimulation [49]. Therefore, according to the Sample Size Calculation, the minimum number of patients in each group, with power value = 95% and expected significance level (*p*-value) = 0.05, should be 13 patients or more.

### 2.3. Clinical Examination

#### 2.3.1. Neurological Examination

A neurological examination was performed by a board-certified neurologist blinded to participant status. Neurological data were recorded in a standardized form, indicating the presence or absence of abnormalities. Anamnesis, physical, and laboratory examination were studied in detail. Mental pathology, cranial nerve abnormalities, cerebellar and gait disorders, motor deficits, changes in reflexes, development of pathological reflexes, and the presence of negative and positive sensory symptoms were examined.

#### 2.3.2. Psychiatric Examination

The diagnosis of GAD was made by a board-certified psychologist and psychiatrist based on the results of a mental and behavioral diagnostic examination. The diagnosis was confirmed if the anxiety state was combined with three or more of the following symptoms for at least 6 months: restlessness, fatigue, difficulty concentrating, muscle tension, sleep disturbances, and irritability. The current level of each symptom was rated on an 11-point scale by the patients themselves.

### 2.4. Anxiety Assessment Tools

#### 2.4.1. Generalized Anxiety Disorder 7-item scale (GAD-7)

Patients independently compiled a questionnaire consisting of seven questions with a choice of one answer out of four: Not at all, Several days, More than half the days, or Nearly every day, which corresponds to 0, 1, 2, or 3 points. The total score in all patients was greater than 4 points and did not exceed 14 points, which corresponds to mild and moderate severity of anxiety.

#### 2.4.2. Hamilton Anxiety Rating Scale

On a 5-point scale, patients self-rated their feelings on 14 anxiety-related items. Each item is scored on a scale from 0 (not present) to 4 (severe), with a total score range of 0–56. The optimal ranges for anxiety on the HAM-A scale were as follows: none or minimal ≤ 7, mild = 8–14; moderate = 15–23; severe ≥ 24. In the examined patients, scores ranged from 8 to 23 points, which corresponds to mild and moderate anxiety, and averaged 15.15 ± 0.31 points.

#### 2.4.3. Quality of Life Enjoyment and Satisfaction Questionnaire

The degree of pleasure and satisfaction experienced by patients during the past week in various areas of daily activities was measured using the Quality of Life, Enjoyment, and Satisfaction Questionnaire—Short Form (Q-LES-Q-SF).

Overall enjoyment and satisfaction with physical health, mood, work, household and leisure activities, social and family relationships, daily functioning, sexual desire, economic status, vision in terms of ability to do work or hobbies, ability to move physically, well-being, TENS treatment, and life contentment was assessed by the patients themselves in 16 items on a 5-point scale. The total score ranges from 16 to 80 and is expressed as a percentage of the maximum total score across all items (0–100). The higher the score, the more pleasure and satisfaction with life. If the results exceed 70%, they are considered normal.

### 2.5. Electroencephalography

To record the bioelectrical activity of the brain, a 16-channel EEG device was used with fixation of active electrodes at Fp1, Fp2, F3, F4, C3, C4, P3, P4, O1, O2, F7, F8, T7, T8, P7, and P8 according to the international 10–20 system and fixation of reference electrodes on the left and right mastoid process. EEG examination was carried out on the EEG-EMG hardware complex—“Neuroexpeditor”, MBN company (registration certificate No. FSR 2010/07889). The electrode impedance did not exceed 5 kOm. The study was conducted in an electrically shielded, dark room. During the study, patients were in quiet wakefulness with closed eyes for 10 min. EEG data were preprocessed by a digital pass filter of a fourth-order Butterworth band. Electromyography, cardiac, pulse, and respiratory artifacts were removed by Independent Component Analysis (ICA). The EEG record was divided into 60 segments. Each segment lasts 10 s. In each EEG segment, the power spectra of theta (4–8 Hz), alpha1 (8–13 Hz), beta I (13–20 Hz), and beta II (20–30 Hz) rhythms were studied [50]. In the analysis, we used two characteristics of EEG:

- Power spectrum density (PSD). PSD is a frequency spectrum, which is calculated using a discrete Fourier transform as the mean squared amplitude of each frequency component [51].
- EEG coherence between occipital and frontal FO regions. EEG coherence is a promising approach for assessing functional cortical connections between different cortical regions. The higher the coherence, the higher the linear synchrony, indicating strong functional connectivity and synergism between different brain regions [52].

It is important to note that ICA, PSD, and EEG coherence are part of the EEG program and are performed when a specific segment is selected. In this work, each EEG study examined 60 segments with a total duration of 10 s. Summary analysis is calculated automatically using several types of mathematical formulas and algorithms.

### 2.6. Transcutaneous Electroneurostimulation

The cathode was fixed at the level of the right wrist above the projection of the entrance of the median nerve into the carpal tunnel. The anode was fixed on the distal part of the middle finger. In sham stimulation, ineffective TENS was used. In the TENS groups, HF-TENS underwent high-frequency TENS with low amplitude, and LF-TENS completed low-frequency TENS with high amplitude (Table 2). The duration of stimulation was 20 min. The number of procedures was 30. The procedures were carried out every day.

**Figure 2:**
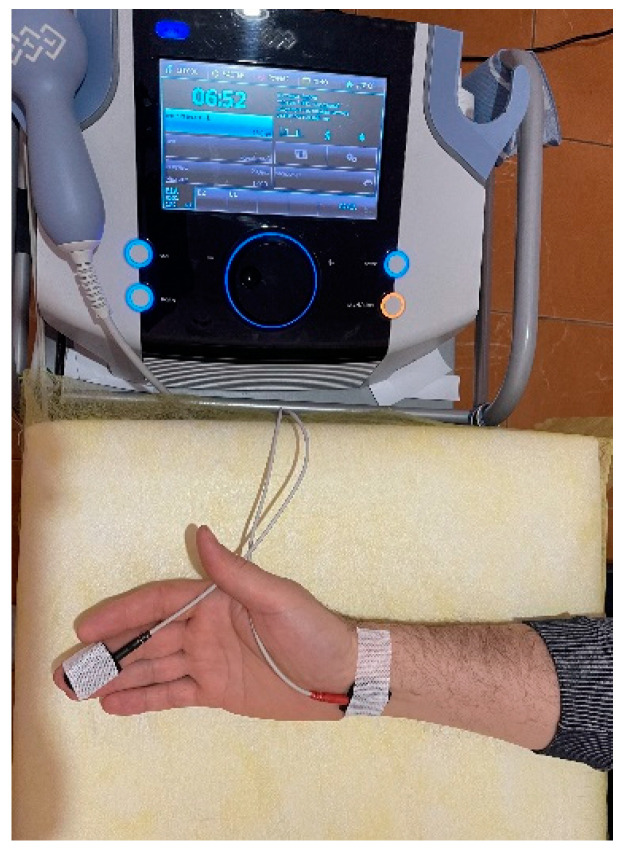
Stimulation of the median nerve by low-frequency, high-amplitude transcutaneous electrical nerve stimulation. The cathode was fixed over the carpal tunnel, and the anode was fixed on the palm distal to the middle finger.

Equipment CE0434 certified BL-4000 smart/premium device (BL Industries Ltd., Hertfordshire, UK) was used for TENS (registration number is RAN 2020/12648, dated 24 November 2020). The equipment has been used in Russia since 2010 (registration number—FEZ 2010/06686, dated 29 April 2010) (Figure 2).

### 2.7. Statistical Analysis

Data analysis was processed using SPSS software for Windows, version 20. Mean (M), standard deviation (SD), and standard error of the mean (SEM) of the participants’ characteristics were calculated. The Shapiro–Wilk test was used to determine normality, and Levene’s test was used to test equality of variances. By multivariate NOVA test, differences between the three groups were tested statistically. The Bonferroni correction test was applied to reduce the chances of obtaining false-positive results (type I errors). Chi-square test was also used to test the significance between two or more categorical groups. To compare the means of the same variable between two groups, we used an independent group t test. The *p* value was set at 0.05.

## 3. Result

### 3.1. Clinical Examination

No symptoms of mental or cognitive disorders were identified in all examined patients. Eye movements were symmetrical within normal limits and there was no nystagmus. No voice or swallowing disorders were found. There were no motor or sensory deficits. Pathological reflexes were not elicited. No gait or coordination disturbances were observed.

#### 3.1.1. Clinical Symptoms of GAD

A study of the six most common complaints in patients with GAD (restlessness, fatigue, difficulty concentrating, muscle tension, sleep disturbances, and irritability) revealed that all these unpleasant disorders occur in almost all patients examined. Even in the absence of any symptoms during medical history collection, patients rated the severity of these symptoms in points when filling out questionnaires.

**Figure 3:**
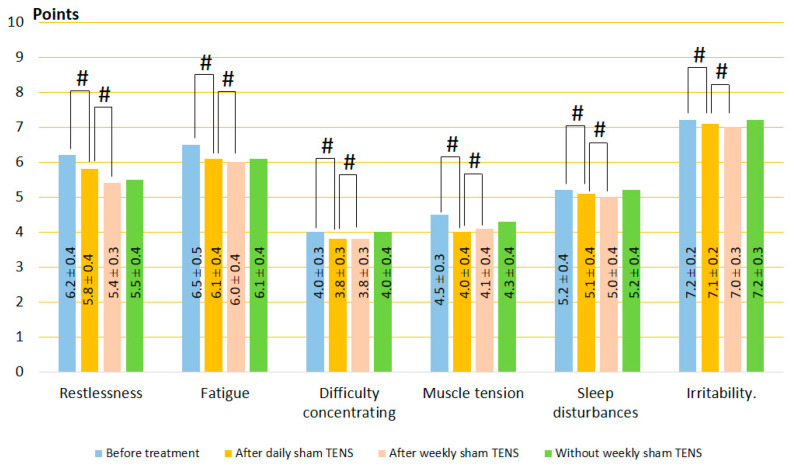
Dynamics of GAD symptoms after treatment in the control group. Note: # *p* > 0.05.

**Figure 4:**
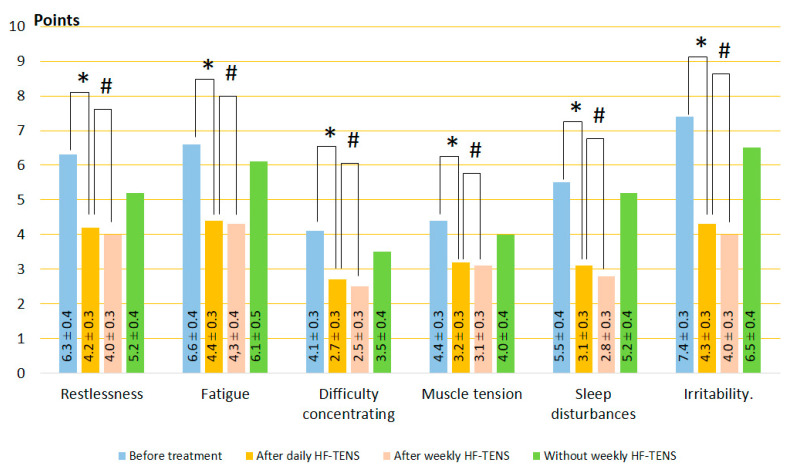
Dynamics of GAD symptoms after treatment in the HF-TENS subgroup. Notes: HF-TENS: high-frequency, low-amplitude transcutaneous electrical nerve stimulation; # *p* > 0.05; * *p* ≤ 0.05.

**Figure 5:**
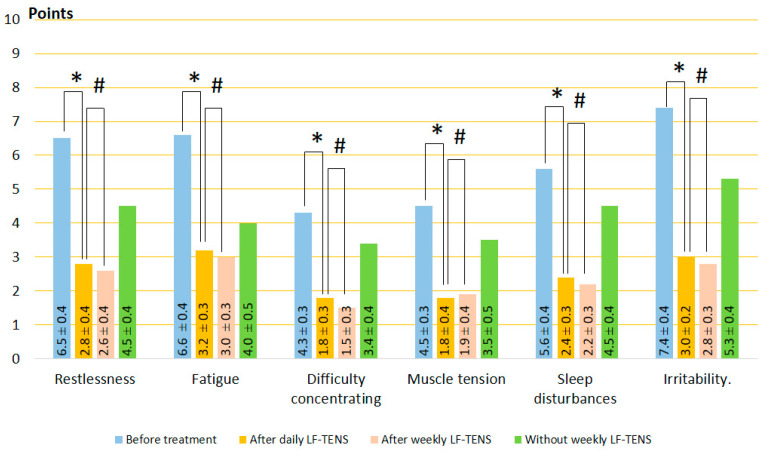
Dynamics of GAD symptoms after treatment in the LF-TENS subgroup. Notes: LF-TENS: low-frequency, high-amplitude transcutaneous electrical nerve stimulation; # *p* > 0.05; * *p* ≤ 0.05.

- Before treatment, self-reported symptoms were irritability, fatigue, and restlessness as the most severe, with an average of 7.33 ± 0.1, 6.56 ± 0.2, and 6.33 ± 0.2, respectively. Difficulty concentrating, muscle tension, and sleep disturbances had a moderate character and averaged 4.67 ± 0.3 points. Results of the study before and after treatment are demonstrated in Figure 3, Figure 4 and Figure 5. The mean of each symptom did not differ significantly between groups.

- After daily TENS, In the control group, no significant changes in GAD symptoms were recorded after sham stimulation (t = 0.60, *p* = 0.55). On the other hand, GAD symptoms decreased to 34% (t = 4.10, *p* = 0.0001) after HF-TENS and to 45% (t = 6.60, *p* = 0.0001) after LF-TENS. Thus, the reduction in GAD symptoms was greater after LF-TENS than HF-TENS by 31.5% (t = 2.30, *p* = 0.024).
- After weekly TENS, after sham stimulation, no changes were detected in all studied symptoms. Continuation of weekly TENS treatment did not improve the results of daily TENS but maintained the same level of improvement over 6 months (*p* > 0.05).
- Follow-up period without weekly TENS, during the first 2 months, GAD symptom scores were not significantly different from those obtained after daily TENS treatment. At the end of the 6-month observation period, the severity of GAD symptoms gradually increased and was greater than after daily TENS in the HF-TENS subgroup by 39.2% (t = 2.86, *p* = 0.005) and in the LF-TENS subgroup by 68% (t = 3.40, *p* = 0.001) and less than the initial values before treatment by 27.6% (t = 3.20, *p* = 0.002) in the LF-TENS subgroup. However, the small residual reduction in GAD symptoms in the HF-TENS group compared with baseline levels (10.9%) was not significant (t = 1.24, *p* = 0.21). The results after sham TENS were the same as before treatment.

#### 3.1.2. Hyperreflexia

Tendon reflexes were increased in 74.4% (*n* = 90) of patients. The knee reflex was examined on both sides, and its intensity was determined in three-level points: normal = 0 points, moderately increased reflex (without increase in the reflexogenic zone) = 1 point, and strongly increased reflex (with increase in the reflexogenic zone) = 2 points. In our study, only patients with identified symmetric hyperreflexia were studied. Statistical processing included averaged values of both sides.

**Figure 6:**
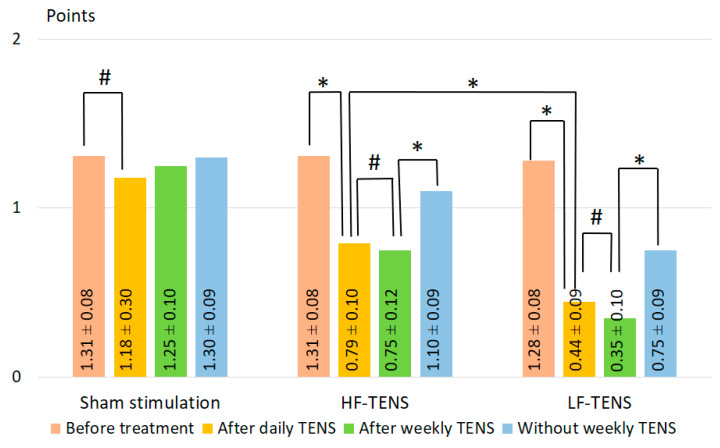
Dynamics of knee hyperreflexia after treatment in the studied groups. Notes: HF-TENS: high-frequency, low-amplitude transcutaneous electrical nerve stimulation; LF-TENS: low-frequency, high-amplitude transcutaneous electrical nerve stimulation; # *p* > 0.05; * *p* ≤ 0.05.

- Before treatment (Figure 6), in all groups, hyperreflexia averaged 1.30 ± 0.05 and did not differ significantly between the study groups (*p* 0 > 0.05).
- After daily TENS, a decrease in hyperreflexia was observed only in patients after treatment with TENS and was not observed after sham stimulation. The reduction in hyperreflexia after HF-TENS averaged 39.3% (t = 4.06, *p* = 0.0002) and after LF-TENS—65.4% (t = 6.89, *p* = 0.0001). Comparative analysis between the two groups showed that hyperreflexia after LF-TENS was lower than after HF-TENS by 44.3% (t = 2.24, *p* = 0.029).
- After weekly TENS, in the subgroups continuing treatment with weekly TENS, the reduction in hyperreflexia remained without significant changes (*p* > 0.05).
- Follow-up period without weekly TENS, in subgroups where weekly TENS was not continued, hyperreflexia remained at the same level in the second month of follow-up as was achieved after daily TENS (*p* > 0.05). At 6 months, there was an increase in hyperreflexia, which decreased from pre-treatment baseline to 16.0% after HF-TENS (t = 1.66, *p* = 0.1) and 41.4% after LF-TENS (t = 4.40, *p* = 0.0001) and was superior to results after daily TENS by 39.2% (t = 2.57, *p* = 0.014) in the HF-TENS subgroup and by 68.5% (t = 2.57, *p* = 0.015) in the LF-TENS subgroup. In addition, after 6 months without treatment, hyperreflexia was lower in the LF-TENS subgroup compared to the HF-TENS subgroup by 31.8%.

### 3.2. Anxiety Assessment Tools

#### 3.2.1. Generalized Anxiety Disorder 7-Item (GAD-7) Scale

**Figure 7:**
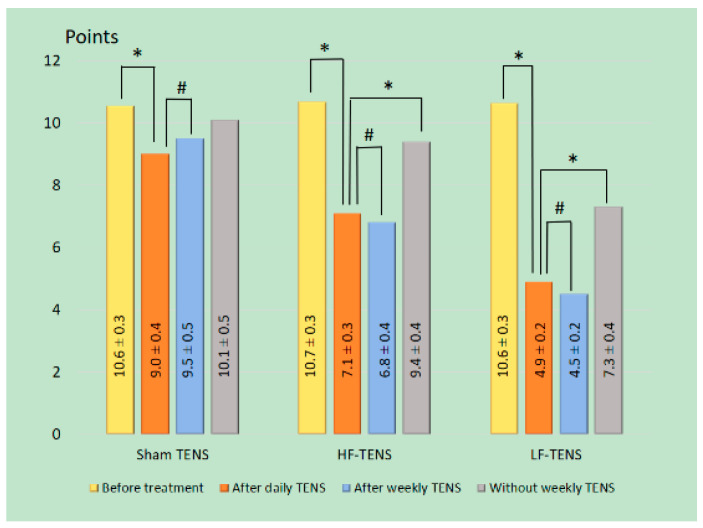
Dynamics of generalized anxiety disorder severity according to the Generalized Anxiety Disorder 7-item before and after treatment in all study groups. Notes: LF-TENS: low-frequency, high-amplitude transcutaneous electrical nerve stimulation; HF-TENS: high-frequency, low amplitude transcutaneous electrical nerve stimulation; # *p* > 0.05 (non-significant difference); * *p* ≤ 0.05 (significant difference).

- Before treatment: Anxiety severity by GAD-7 averaged 10.6 ± 0.18 points (Figure 7) with no significant differences between groups (*p* > 0.05).
- After daily treatment with sham TENS, a slight decrease in anxiety was noted, which did not exceed 15.1% (t = 3.20, *p* = 0.002). After effective TENS, the regression of anxiety was higher than in the control group by 21.1% (t = 3.20, *p* = 0.002) in the HF-TENS subgroup and by 45.6% (t = 3.20, *p* = 0.002) in LF-TENS subgroup. Additionally, the anxiolytic properties of LF-TENS were found to be more effective than HF-TENS by 59.8% (t = 5.50, *p* = 0.001).
- In patients continuing treatment with weekly TENS, there were no significant dynamics compared with the level of anxiety after daily treatment of TENS by sham TENS, HF-TENS, or LF-TENS (*p* > 0.05).
- The follow-up period of patients who did not undergo weekly TENS revealed that in the first 2 months, the severity of anxiety did not differ significantly from the values obtained after daily TENS in all groups. In subsequent months, a gradual increase in anxiety was noted, reaching a maximum at the end of the sixth month of observation. Compared to daily TENS results, there was an increase in the control group by 12% (t = 1.72, *p* = 0.09), in the HF-TENS subgroup by 32% (t = 4.60, *p* = 0.0001), and in the LF-TENS subgroup by 49% (t = 5.36, *p* = 0.001). However, the severity of anxiety was less than the initial values before treatment in the HF-TENS subgroup by 33% (t = 2.60, *p* = 0.01) and in the LF-TENS subgroup by 34% (t = 6.60, *p* = 0.0001) and did not differ in the control group (t = 0.86, *p* = 0.39). Thus, the anxiolytic effect of daily LF-TENS had a more sustained effect compared to HF-TENS by 1.6 times for at least 6 months.

#### 3.2.2. Hamilton Anxiety Rating Scale

**Figure 8:**
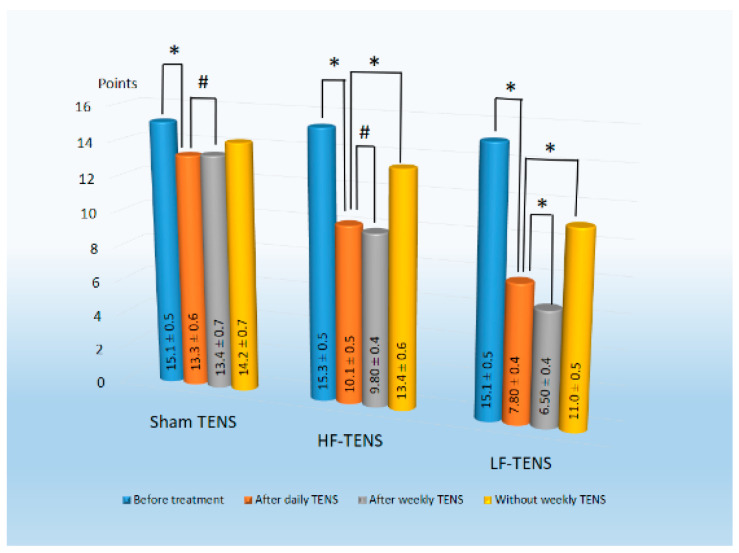
Dynamics of generalized anxiety disorders severity according to the Hamilton Anxiety Rating Scale before and after treatment in all study groups. Notes: LF-TENS: low-frequency, high-amplitude transcutaneous electrical nerve stimulation; HF-TENS: high-frequency, low-amplitude transcutaneous electrical nerve stimulation; # *p* > 0.05 (non-significant difference); * *p* ≤ 0.05 (significant difference).

- Before treatment, when studying anxiety disorders using the HAM-A method, it was revealed that the severity of GAD symptoms averaged 15.1 ± 0.3 points (Figure 8). No considerable differences were recorded between the mean values of the study groups (*p* > 0.05).
- After daily TENS treatment, the reduction in anxiety disorders using sham TENS, HF-TENS, and LF-TENS averaged 11.9% (t = 2.30, *p* = 0.023), 34.0% (t = 4.09, *p* = 0.0001), 48.3% (t = 7.04, *p* = 0.0001), respectively. Apparently, the anxiolytic effect of effective TENS was more pronounced than that of sham TENS, which reached 24.1% (t = 4.10, *p* = 0.0001) after HF-TENS and 41.4% (t = 2.30, *p* = 0.023) after LF-TENS. From the results obtained, it can be seen that the reduction in anxiety disorders after LF-TENS is 42.2% (t = 3,59, *p* = 0.0006) higher compared to HF-TENS.
- The results of continued treatment with weekly TENS showed that the anxiolytic effect obtained after daily TENS remained at the same level after using sham TENS and HF-TENS (*p* > 0.05) and significantly improved after using LF-TENS by 16.6% (t = 2.29, *p* = 0.024).
- Observation of patients who did not undergo weekly maintenance TENS revealed that the positive effect after daily TENS was maintained during the first 2 months. However, at the end of the follow-up period, scores obtained after daily therapy worsened by 32% (t = 4.20, *p* = 0.0001) after HF-TENS and by 41% (t = 5.62, *p* = 0.0001) after LF-TENS. Despite this, the severity of anxiety was lower than the initial values before treatment by 12.4% (t = 2.43, *p* = 0.02) after HF-TENS and by 27.2%(t = 5.66, *p* = 0.0001) after LF-TENS.

#### 3.2.3. Quality of Life Enjoyment and Satisfaction Questionnaire

**Figure 9:**
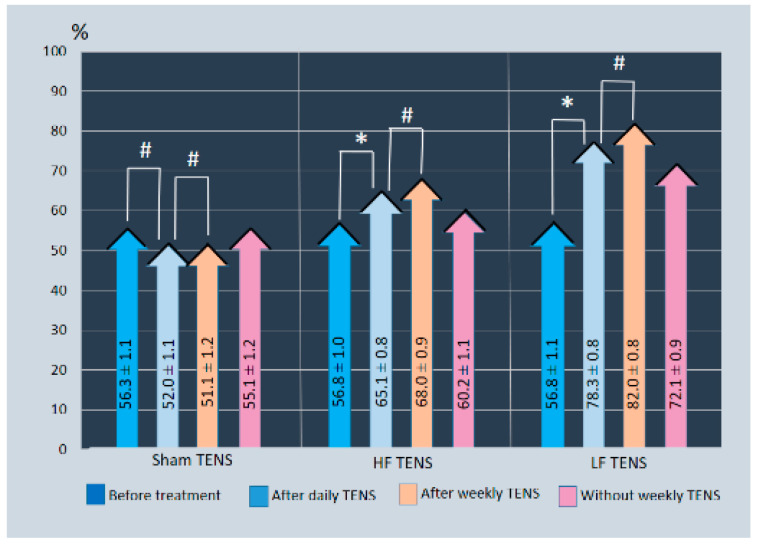
Dynamics of Quality of Life Enjoyment and Satisfaction Questionnaire scores before and after treatment in all study groups. Notes: LF-TENS: low-frequency, high-amplitude transcutaneous electrical nerve stimulation; HF-TENS: high-frequency, low-amplitude transcutaneous electrical nerve stimulation; # *p* > 0.05 (non-significant difference); * *p* ≤ 0.05 (significant difference).

- Quality of life enjoyment and satisfaction on average in all groups was 56.6 ± 0.62% without noticeable differences between them (Figure 9).
- After daily TENS, the improvement in quality of life increased by 14.6% (t = 3.27, *p* = 0.002) in the HF-TENS subgroup and by 37.9% (t = 3.27, *p* = 0.002) in the LF-TENS subgroup. No significant change was found in the control group. Thus, the quality of life after LF-TENS was 1.59 times (t = 11.6, *p* = 0.0001) higher than after HF-TENS (t = 3.27, *p* = 0.002).
- The quality of life enjoyment and satisfaction after a weekly TENS remained at the same level as after completing a course of daily TENS.
- Without continuing the course of weekly TENS, the quality of life of patients compared to the level before treatment was at the same level in the control group and exceeded this level by 6.0% (t = 2.29, *p* = 0.02) in the HF-TENS subgroup and by 26.9% (t = 10.7, *p* = 0.0001) in the LF-TENS subgroup.

### 3.3. Electroencephalography

Sixty artifact-free 10 s EEG segments of each EEG study were acquired and calculated using PSD and coherence determination.

#### 3.3.1. Power Spectrum Density

**Figure 10:**
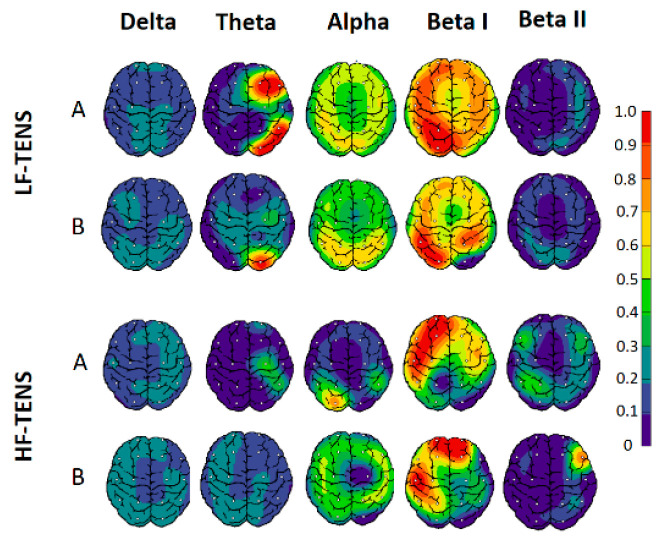
Dynamics of power spectrum density of delta, theta, alpha, beta I, and beta II rhythms before (A) and after weekly TENS (B) in all study groups. Notes: LF-TENS: low-frequency, high-amplitude transcutaneous electrical nerve stimulation; HF-TENS: high-frequency, low-amplitude transcutaneous electrical nerve stimulation.

- Before treatment, PSD analysis revealed significant changes in theta, alpha, beta I, and beta II rhythms in the frontal, central, parietal, temporal, and occipital regions. More significant changes were associated with a decrease in the PSD of the alpha rhythm in the occipital regions with an increase in the PSD of the beta I and beta II rhythms in the frontal, central, and temporal regions (Figure 10). The changes were individual in nature: from a moderate increase in the beta rhythm in the temporal, central, and frontal regions to hypersynchronization of the high-amplitude beta rhythm in all regions. However, the most frequent increases in beta I and beta II PSD were localized in the left hemisphere, with markedly greater amplitudes in the temporal regions (Figure 5).
- After daily TENS, no significant changes were detected in all groups, regardless of the treatment method.
- After weekly TENS, EEG showed a significant improvement in EEG characteristics with an increase in PSD for alpha rhythm in occipital regions by 24%(t = 2.09, *p* = 0.04) and a decrease in PSD for beta I rhythm in temporal and frontal regions by 28% (t = 2.14, *p* = 0.036) in patients after LF-TENS. EEG changes after HF-TENS and sham TENS were unremarkable.
- At the end of the follow-up period in patients who did not receive weekly TENS, PSD was not significantly different from other baseline EEG findings in all study groups.

#### 3.3.2. EEG Coherence

**Figure 11:**
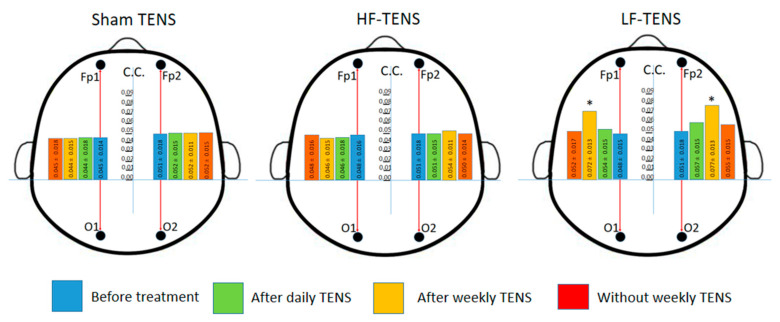
Dynamics interhemispheric coefficient coherence (C.C.) between frontal (Fp1-cлeвa and Fp2-cпpaвa) and occipital (O1-cлeвa and O2-cпpaвa) regions of alpha rhythm before and after treatment in all study groups. Notes: LF-TENS: low-frequency, high-amplitude transcutaneous electrical nerve stimulation; HF-TENS: high-frequency, low-amplitude transcutaneous electrical nerve stimulation; * *p* ≤ 0.05.

- The values of cross-regional frontal coherence ranged from 0.3 to 0.7 with an average of 0.4 ± 0.01(normal range for adults 0.4–0.6). Meanwhile, coherence analysis showed that there were no statistical differences between the groups either before or after treatment. The determination of the cross-regional coherence coefficient between other regions was not meaningful due to the initial marked differences between groups.
- Before treatment, F-O interhemispheric coherence was significantly weak in all patients, ranging from 0.01 to 0.11, with a mean of 0.046 ± 0.01 in the left hemisphere and 0.051 in the right hemisphere (normal range for adults 0.14–0.16).
- After daily TENS, no significant influence on F-O interhemispheric coherence was found for sham TENS, HF-TENS, or LF-TENS (*p* > 0.05).
- Treatment continued for 6 months with weekly TENS did not significantly change the baseline coherence coefficient value in the sham TENS group or the HF-TENS subgroup. Only after LF-TENS, the relationship between the interhemispheric frontal and occipital regions was recorded with a significant increase in the coherence coefficient by 93% (t = 2.09, *p* = 0.040) in the left hemisphere and by 74.5% (t = 2.03, *p* = 0.046) in the right hemisphere (Figure 11).

## 4. Discussion

One hundred and twenty-one patients who met the GAD-7 and HAM-A diagnostic criteria for GAD were recruited from the Clinic of Vertebral and Brain Diseases and assessed by neurologists and psychiatrists using the Structural Clinical Interview for DSM-V Disorders. The severity of GAD in patients ranged from mild to moderate. All patients included in our study did not receive pharmacotherapy due to the lack of effect from its use in the past or the development of side effects. Among the negative consequences of pharmacotherapy, patients noted cognitive impairment (sedation, drowsiness, and mental slowing), sexual disorders (erectile dysfunction and decreased libido), and the inability to drive safely. The control group underwent sham TENS, and the treatment group underwent effective TENS, which was divided according to TENS characteristics into the HF-TENS subgroup and LF-TENS subgroup.

Our study consisted of two stages: In the first stage, TENS was performed daily for 30 days. In the second stage, half of the patients continued maintenance treatment with TENS once a week for 6 months (Weekly TENS), and the other half did not continue treatment and were under our observation for 6 months.

### 4.1. Severity of GAD before Treatment

Self-reported irritability, fatigue, restlessness, difficulty concentrating, muscle tension, and sleep disturbances were moderate on average, which corresponded to the results of assessing the severity of anxiety disorders on the GAD-7 and HAM-A scales. Hyperreflexia was detected in the majority of patients. Deep tendon reflexes were generally symmetrical laterally, but the knee reflex was the most hyperactive. The results of the EEG study indicate dysfunction of the cerebral cortex in most parts of the brain in all patients. Nevertheless, these changes were individual in nature. However, the increase in beta rhythm appeared to be a common pattern in all cases, most prominently in the frontal, central, and temporal regions, with a clear predominance over the left hemisphere. Moreover, more severe GAD was associated with the involvement of more brain regions and higher beta rhythm in temporal regions. Reduction in F-O interhemispheric coherence was registered in all patients.

### 4.2. Severity of GAD after Treatment by TENS

Daily use of effective TENS for a month resulted in a reduction in symptoms of GAD by 39.5% (*p* ≤ 0.05), hyperreflexia by 52.35% (*p* ≤ 0.05), GAD-7 scores by 59.8% (*p* ≤ 0.05), and HAM-A scores by 41.1%. After sham TENS, a mild decrease in the severity of GAD was recorded according to GAD-7 and HAM-A by an average of 13.5% (*p* ≤ 0.05). A comparative analysis revealed a high efficiency of LF-TENS over HF-TENS by 45% (*p* ≤ 0.05), a reduction in GAD symptoms by 34% (*p* ≤ 0.05), a normalization of knee reflex by 45% (*p* ≤ 0.05), and a decrease in GAD severity by 51.03% (*p* ≤ 0.05). The logical consequence was an increase in the quality of life enjoyment and satisfaction of patients after effective TENS by 26.2% (*p* ≤ 0.05) against the background of regression of the severity of GAD with a more pronounced effect by 1.6 times after LF-TENS (*p* ≤ 0.05). No significant changes were recorded in EEG activity or brain functional connectivity. Continuation of treatment for 6 months using weekly TENS prolonged the achieved positive effect in all groups without significant changes and contributed to the improvement of EEG activity. Only after long-term treatment with LF-NENS, a decrease in beta rhythm PSD in the temporal and frontal regions and an increase in alpha rhythm PSD in the occipital regions were recorded. The functional connectivity of the brain between the frontal and occipital regions increased noticeably (interhemispheric coherence of the FO) by 83.8% (*p* ≤ 0.05). However, in patients who did not continue treatment with weekly TENS, the results obtained after daily TENS remained without significant negative dynamics during the first 2 months of the follow-up period in all groups. However, by the end of the sixth month of the follow-up period, the severity of GAD symptoms, as well as GAD-7 and HAM-A scores, increased in all groups and remained at pre-treatment baseline levels in patients receiving sham TENS and below this level in patients after LF-TENS and HF-TENS by 31.9% (*p* ≤ 0.05) and 12.9% (*p* > 0.05), respectively. Additionally, quality of life returned to baseline levels in patients completing sham TENS and HF-TENS and continued to be 26.9% (*p* ≤ 0.05) higher than baseline levels in patients undergoing LF-TENS.

### 4.3. Pathogenesis of the Anxiolytic Effect of TENS

Neurological hyperactivity in GAD is related to stress–response hyperstimulation, which develops to increase the body’s defense capability to real danger factors [53]. However, in individuals with moderate adaptational reserve, these changes can become pathological [54] with the development of irritability, muscle tension, and sleep disturbances associated with aberrant regional brain activity in areas connected with emotion processing [55]. Moreover, the overactivity of the amygdala and insula may lead to “misinterpretation” of ambiguous stimuli as a threat [56,57]. In contrast, the depletion of resistance is the result of a prolonged ineffective fight against chronic, unattenuated harmful factors and can be crucial for the development of neurotransmitter imbalance [58]. Gamma-aminobutyric acid (GABA) plays a central role in the pathophysiology of anxiety disorders, which has a major inhibitory effect on neuronal excitability [59]. Baseline anxiety scores on the HAM-A were positively correlated with GABA levels measured using proton magnetic resonance spectroscopy (1H-MRS) in patients with GAD [60] and concentrated in cortical and limbic areas [61]. Additionally, experimentally it was found that mice with high anxious behavior have higher levels of GABA in the amygdala and greater expression of the GABA pathway components GAD65 and GAD67 than mice with normal anxious behavior [62]. Nevertheless, monitoring GABA levels over time using 1H-MRS has shown that GABA levels can change without concomitant changes in anxiety symptoms, suggesting that symptom profiles in GAD are complex and multifactorial [60]. Obviously, all these changes on the neural level cannot occur without the development of EEG disturbances in patients with GAD. Increased beta PSD in GAD has been reported in many studies [63,64], which can be explained by the vigilant and excited state of the brain, [65,66] and mediates higher cognitive functions [67,68]. Scattered studies have investigated and found that weak coherence is associated with abnormal electrophysiological disconnectivity between brain regions in patients with GAD [69].

### 4.4. Efficiency of TENS in the Treatment of GAD

Previous studies have demonstrated the effectiveness of high-frequency and low-frequency TENS in increasing GABA levels and activating GABA(A) receptors in the spinal cord [70,71,72] and brain [73]. The effects of TENS also extend to other neurotransmitters, such as endogenous central endorphins, which increase primarily after LF-TENS [74]. Additionally, the high effectiveness of TENS on central structures that play a decisive role in the development of anxiety disorders has been confirmed in many studies using fMRI. Following TENS, there was a decrease in BOLD signal in limbic brain regions, including the amygdala, hippocampus, parahippocampal gyrus, middle, and superior temporal gyrus, which was accompanied by increased activation of the insula, precentral gyrus, and thalamus [75,76]. The anxiolytic effect of TENS has been found in the treatment of patients with insomnia [77,78], distal polyneuropathy [27], carpal tunnel syndrome [26], post-traumatic stress disorder [79], post-hemorrhoidectomy syndrome [80], in preoperative anxiolytic preparation before thoracoscopic surgery [81], during induction of labor [44], and dental procedures [82]. However, in rare studies, TENS has been successfully used in the treatment of GAD [29]. LF-TENS is known in the literature as an acupuncture-like TENS [83]. Recently, there has been growing interest among researchers in studying TENS of Daling (PC7), acupuncture points of the pericardial meridian. Anatomically, this point is located above the median nerve in the carpal tunnel [84]. Electrical stimulation of these acupuncture points improves functional connections between brain regions associated with normalizing emotions by 24.9% [78,85,86]. The anxiolytic effect results from activation of the prefrontal cortex, insula, temporal gyrus, anterior cingulate cortex, temporal pole, dorsolateral prefrontal cortex, and inferior frontal cortex, which are closely associated with cognition, spirit, and emotion in the brain [86]. Contemporary studies have shown a significant effect of TENS intervention on cortical activity with suppression of sensory evoked potentials and increase in PSD of theta and alpha rhythms [87] and in the improvement of functional brain connectivity at both regions and network levels [41,88]. In other studies after TENS intervention, a significant improvement in corticospinal and motor cortex excitability was noted, as well as an increase in the volume of the motor cortical map [89]. There are similar works in the literature on stimulation of the median nerve in the treatment of patients with high blood pressure. As a result of this work, there was a decrease in sympathetic influence and normalization of heart rate and blood pressure. It is possible that this result is of a central nature due to the normalization of the autonomic nervous system [43]. Recently, the use of auricular transcutaneous vagus electrical stimulation has become widespread in the treatment of many diseases, including GAD and depression. However, results vary and the mechanisms of action are not entirely clear [39,42,48]. Further comparative studies of median and vagus nerve stimulation in the treatment of GAD are needed.

## 5. Conclusions

According to the results of our study, it has been proven that 30 days of HF-TENS and LF-TENS compared to sham TENS are more effective in the treatment of GAD, leading to a significant prolonged improvement in clinical manifestations of GAD as a minimum of 2 months. However, LF-TENS is superior in effectiveness to HF-TENS and only leads to the recovery of neurophysiological disorders on the EEG. The prolonged effect of HF-TENS and LF-TENS was maintained without negative dynamics when TENS treatment was continued weekly throughout the entire 6-month observation period. Thus, to maintain a constant therapeutic effect for a longer period, it is recommended to continue treatment with maintenance procedures once a week. Eventually, minimal side effects, low cost, safety, and simplicity of TENS procedures are appropriate as a home or self-treatment modality for a wide range of patients. To meet this target, it is important to produce TENS devices with simple biofeedback mechanisms, battery-powered, affordable, and easy to use.

## 6. Declaration of Patient Consent

The authors confirm that they have obtained all necessary patient consent forms. In the form, the patients gave their consent for the publication of their images and other clinical information in the journal. The patients understand that their names and initials will not be published and appropriate steps will be taken to conceal their identity, but anonymity cannot be guaranteed.

## Figures and Tables

**Table 1:** Demographic and clinical characteristics of the participants.

	Control Group	TENS Group		
		HF-TENS Subgroup	LF-TENS Subgroup	*p*
No	40	40	41	
Age	46.4 ± 1.18	45.8 ± 1.12	46.2 ± 0.98	*p* > 0.05
Gender (female:male)	22:18	21:19	20:21	*p* > 0.05
Disease duration (months)	18.2 ± 1.27	19.2 ± 1.37	18.6 ± 1.24	*p* > 0.05
GAD-7	10.6 ± 0.33	10.7 ± 0.28	10.6 ± 0.32	*p* > 0.05
HAM-A	15.1 ± 0.51	15.3 ± 0.51	15.1 ± 0.53	*p* > 0.05

Note: GAD-7: Generalized Anxiety Disorder 7-item scale; HAM-A: Hamilton Anxiety Rating Scale; HF-TENS: high-frequency, low-amplitude transcutaneous electroneurostimulation; LF-TENS: low-frequency, high-amplitude transcutaneous electroneurostimulation; *p*: level of marginal significance.

**Table 2:** Characteristics of the applied current in different groups.

	Sham Stimulation	HF-TENS	LF-TENS
Waveform	Monophasic square wave	Monophasic square wave	Monophasic square wave
Frequency	1 Hz	50 Hz	1 Hz
Duration	30 mcs	50 mcs	200 mcs
Amplitude *	Minimal sensory response	Distinct painless sensory response	Distinct painless motor response

Note: HF-TENS: high-frequency, low-amplitude transcutaneous electroneurostimulation; LF-TENS: low-frequency, high-amplitude transcutaneous electroneurostimulation; Hz: hertz; mcs: microseconds. * The amplitude was increased from zero until the required response was achieved.

## Data Availability

Data is not available due to confidentiality and ethical restrictions.
